# Characterization of the Skeletal Muscle Secretome Reveals a Role for Extracellular Vesicles and IL1α/IL1β in Restricting Fibro/Adipogenic Progenitor Adipogenesis

**DOI:** 10.3390/biom11081171

**Published:** 2021-08-08

**Authors:** Simone Vumbaca, Giulio Giuliani, Valeria Fiorentini, Flavia Tortolici, Andrea Cerquone Perpetuini, Federica Riccio, Simona Sennato, Cesare Gargioli, Claudia Fuoco, Luisa Castagnoli, Gianni Cesareni

**Affiliations:** 1 Department of Biology, University of Rome Tor Vergata, Via delle Ricerca Scientifica 1, 00133 Rome, Italy; g.giulianigiulio@gmail.com (G.G.); valy.fiore.26@gmail.com (V.F.); flavia.tortolici@hotmail.it (F.T.); andrea.cerquoneperpetuini@gmail.com (A.C.P.); federicariccio1989@libero.it (F.R.); cesare.gargioli@uniroma2.it (C.G.); castagnoli@uniroma2.it (L.C.); cesareni@uniroma2.it (G.C.); 2 Sapienza Unit, Physics Department, Institute for Complex Systems (ISC), National Research Council, Sapienza University of Rome, P.le A. Moro 2, 00185 Rome, Italy; simona.sennato@gmail.com

**Keywords:** muscle regeneration, secretome, cytokine, extracellular vesicles, faps, adipogenesis

## Abstract

Repeated mechanical stress causes injuries in the adult skeletal muscle that need to be repaired. Although muscle regeneration is a highly efficient process, it fails in some pathological conditions, compromising tissue functionality. This may be caused by aberrant cell–cell communication, resulting in the deposition of fibrotic and adipose infiltrates. Here, we investigate in vivo changes in the profile of skeletal muscle secretome during the regeneration process to suggest new targetable regulatory circuits whose failure may lead to tissue degeneration in pathological conditions. We describe the kinetic variation of expression levels of 76 secreted proteins during the regeneration process. In addition, we profile the gene expression of immune cells, endothelial cells, satellite cells, and fibro-adipogenic progenitors. This analysis allowed us to annotate each cell-type with the cytokines and receptors they have the potential to synthetize, thus making it possible to draw a cell–cell interaction map. We next selected 12 cytokines whose receptors are expressed in FAPs and tested their ability to modulate FAP adipogenesis and proliferation. We observed that IL1α and IL1β potently inhibit FAP adipogenesis, while EGF and BTC notably promote FAP proliferation. In addition, we characterized the cross-talk mediated by extracellular vesicles (EVs). We first monitored the modulation of muscle EV cargo during tissue regeneration. Using a single-vesicle flow cytometry approach, we observed that EVs differentially affect the uptake of RNA and proteins into their lumen. We also investigated the EV capability to interact with SCs and FAPs and to modulate their proliferation and differentiation. We conclude that both cytokines and EVs secreted during muscle regeneration have the potential to modulate adipogenic differentiation of FAPs. The results of our approach provide a system-wide picture of mechanisms that control cell fate during the regeneration process in the muscle niche.

## 1. Introduction

Tissue growth and regeneration are governed by stem cell differentiation under the control of the tissue microenvironment [1]. Stromal support-cells, extracellular matrix, and soluble factors are essential components of the stem cell niche and contribute to modulate stem cell fate [2]. The cross-talk within the niche is mediated by cell–cell contacts and by soluble factors, henceforth called “the secretome”. Proteins released as soluble cytokines or enclosed in extracellular vesicles (EVs) [3] are the main component of the secretome [4]. Secretome composition varies in time and space and its dysregulation is associated with the development and progression of several diseases, including pulmonary diseases, rheumatoid arthritis, interstitial fibrosis, and atherosclerosis [5,6,7,8].

The muscle stem cell niche has been extensively investigated since the discovery of its residing stem cell, the satellite cell (SC) [9]. The dynamics of the interactions within this niche account for the striking regeneration capability of the skeletal muscle after acute injury [10]. The injection of cardiotoxin, a myotoxin derived from snakes [11], induces massive myofiber necrosis and activates quiescent SCs. The activation of SCs is followed by proliferation and differentiation to replace the damaged muscle fibers [12]. This process is orchestrated by a complex network of interactions between stem cells, stromal cells, and their secretomes [13]. Among these cell players, fibro/adipogenic progenitors (FAPs) play an essential role in muscle regeneration [14]. Although the role of FAPs during regeneration has not been fully explored, among their known functions, following damage FAPs mainly (i) are activated by IL-4 and IL-13, (ii) promote muscle regeneration by secreting WISP1, IGF-1, and IL-6, (iii) facilitate debris clearance, (iv) contribute to the reconstitution of the Extra-Cellular Matrix (ECM), (v) recruit T regulatory cells, and (vi) stabilize newly formed vessels [15,16,17,18,19,20,21,22]. However, in addition to their pro-regeneration role, in pathological conditions, FAPs are responsible for the development of ectopic intramuscular adipose (IMAT) or connective tissues (IMCT) [16,18,19,23]. Several molecular cues have been reported to be involved in the modulation of FAP differentiation in vitro [15,24,25,26,27,28,29,30]. However, their relative importance in physiology and pathology remains to be established.

The role of EVs in muscle regeneration is less explored. Myoblast- and myotube-derived EVs carry growth factors and miRNAs (MyoMirs) that have been implicated in controlling myogenesis [31,32]. These molecular messages are sensed by myogenic progenitors that respond by modulating both proliferation and differentiation [33,34]. The functional relevance of the cross-talk mediated by EVs is argued by the observation that after treatment with HDAC inhibitors, EVs released by FAPs transfer pro-myogenic microRNA to SCs [35] while EVs produced after a low-load blood flow restricted resistance exercise (BFRE) induced FAP proliferation [36]. In addition, mesenchymal stem cell-derived EVs promote muscle regeneration [37,38]. All these results suggest that EVs may be involved in additional mechanisms, playing multiple roles in the physiology and pathology within the muscle niche.

Prompted by these considerations, we set out to investigate the composition of the skeletal muscle secretome during regeneration induced by cardiotoxin injury. By a multiplex ELISA array, we first defined the expression kinetics of 76 secreted proteins in the 12 days following cardiotoxin injection. In addition, we purified EVs produced within the tissue at days 1, 3, and 5 after injury and characterized their cargo and its variation in composition during muscle regeneration. Finally, we characterized the potential of a selected subset of cytokines and of extracellular vesicles to modulate differentiation and proliferation of FAPs and SCs.

## 2. Materials and Methods

### 2.1. Mouse Model

Adult (3-6 months old) wild-type (wt) C57BL/6 mice purchased from Jackson Laboratories were used in all the experiments. Mice were maintained according to the standard animal facility procedures, and experiments on animals were conducted according to the rules of good animal experimentation (I.A.C.U.C. no. 432 of 12 March 2006).

Prior to cardiotoxin injection, mice were anesthetized with an intramuscular injection of physiologic saline (10 mL/kg) containing ketamine (5 mg/mL) and xylazine (1 mg/mL). 10μM of cardiotoxin (Latoxan, Portes-lès-Valence, France, L81-02) was administered intramuscularly into the tibialis anterior, quadriceps, and gastrocnemius. All experimental protocols were approved on 23/10/2017 by the internal Animal Research Ethical Committee according to the Italian Ministry of Health regulation (protocol #820/2017-PR).

### 2.2. Mononuclear Fraction Isolation and MACS Separation Procedure

Dissected muscles from uninjured and cardiotoxin-injured wt mice were minced mechanically and then dissociated enzymatically for 1 h at 37 °C. The enzymatic mix, diluted in D-PBS with calcium and magnesium, contained 2 μg/mL collagenase A, 2.4 U/mL dispase II, and 0.01 mg/mL DNase I.

The digestion reaction was blocked by diluting in ice cold Hanks’ balanced salt solution with calcium and magnesium (HBSS Gibco, Waltham, MA, United States, Cat. No. 14025-092) supplemented with 0.2% BSA and 1% penicillin–streptomycin (P/S, 10,000 U/mL). In order to remove tissue debris, the cells suspension was subjected to sequential filtrations through 100-, 70-, and 40- μm cell strainers and centrifuged at 600 g for 5 min after each filtration step. Red blood cells (RBC) were lysed using 10× RBC lysis buffer (Santa Cruz Biotechnology, Dallas, TX, United States, Cat. No. sc-296258) diluted to 1x in pre-filtered ddH_2_O. Finally, the cell suspension was filtered with 30 μm cell strainers, obtaining the skeletal muscle mononuclear fraction.

If needed, single cell populations were sorted using the magnetic-activated cell sorting (MACS) separation technology. Starting from the mononuclear fraction, we sequentially sorted: Immune cells using anti-CD45 (Miltenyi, Bergisch Gladbach, Germany, Cat. No. 130-052-301), endothelial cells using anti-CD31 (Miltenyi, Cat. No. 130-097-418), Satellite Cells using anti-ITGA7 (Miltenyi, Cat. No. 130-104-261), and FAPs using anti-Sca1 (Miltenyi, Cat. No. 130-106-641).

### 2.3. Multiplex ELISA Array

Freshly isolated mononuclear cells were pelleted at 800 g for 10 min and washed 3 times with sterile PBS. Cells were lysed in a lysis buffer (RayBiotech, Peachtree Corners, GA, United States, Cat. No. AA-LYS-10mL) supplemented with 1x Protease Inhibitor Cocktail (Sigma-Aldrich, St. Louis, MO, United States, Cat. No. P8340), 1× Phosphatase Inhibitor Cocktail 2 (Sigma-Aldrich, Cat. No. P5726), 1× Phosphatase Inhibitor Cocktail 3 (Sigma-Aldrich, Cat. No. P0044), and incubated in ice for 30 min. The lysates were centrifuged at 15,000× *g* at 4 °C for 30 min and stored at −80 °C. Cell lysates were sent to the tebu-bio facility in order to perform Multiplex ELISA array and subsequent technical normalizations.

### 2.4. Cell Culture and Treatments

Freshly isolated FAPs were cultured at 37 °C and 5% CO_2_ in Growth Medium (GM) containing Dulbecco’s Modified Eagle’s Medium (DMEM) and supplemented with 20% heat-inactivated Fetal Bovine Serum (FBS), 100 U/mL penicillin–streptomycin, 1mM sodium pyruvate, and 10mM HEPES.

To induce FAPs adipogenesis, after 3 days in GM, FAPs were exposed to Adipogenic Induction Medium (AIM) which consisted of DMEM supplemented with 20% heat-inactivated FBS, 0.5 mM 3-isobutyl-1-methylxanthine (Sigma-Aldrich, Cat. No. I5879), 0.4 mM dexamethasone (Sigma-Aldrich, Cat. No. D4902), 1 μg/mL insulin (Sigma-Aldrich, Cat. No. I9278), 100 U/mL penicillin–streptomycin, 1 mM sodium pyruvate, and 10 mM HEPES. After 3 days in AIM, FAPs were switched for 3 days to Maintenance Medium (MM) composed of DMEM with 10% heat-inactivated FBS, 1 μg/mL insulin, 100 U/mL penicillin–streptomycin, 1 mM sodium pyruvate, and 10 mM HEPES.

To perturb FAPs fibrogenesis, after 2 days in GM, FAPs were exposed to GM supplemented with 1 ng/mL of TGFb for 5 days.

Freshly isolated SCs were cultured at 37 °C and 5% CO_2_ on Matrigel-coated dish with Growth Medium (sGM), composed of DMEM supplemented with 20% heat-inactivated FBS, 10% heat-inactivated horse serum (HS), 2% chicken embryo extract (CEE), 100 U/mL penicillin–streptomycin, 1 mM sodium pyruvate, and 10 mM HEPES. After 3 days in GM, SCs were switched in Differentiation Medium (sDM) composed of DMEM supplemented with 2% heat-inactivated HS, 100 U/mL penicillin–streptomycin, 1mM sodium pyruvate, and 10 mM HEPES.

To perturb FAPs proliferation with muscle-derived EVs, cells were seeded in DMEM supplemented with 20% exosome-depleted heat-inactivated FBS (Gibco™ A2720803), 100 U/mL penicillin–streptomycin, 1 mM sodium pyruvate, 10 mM HEPES, and 4000 EVs/µL. Instead, to perturb differentiation process with muscle-derived EVs, cells were seeded in GM using 20% exosome-depleted heat-inactivated FBS (Gibco™ A2720803) instead of usual FBS supplemented with 4000 EVs/µL. After 3 days in this modified GM, cells were switched in AIM with exosome-depleted heat-inactivated FBS instead of usual FBS, supplemented with 4000 EVs/µL. To perturb FAPs differentiation with cytokines, cells were treated along all the in vitro differentiation protocol that consisted of 3 days in GM, 3 days in AIM, and 3 days in MM.

To perform the growth curve experiment, 2000 FAPs were seeded in GM in 96-multiwell plates (6000 cells/cm^2^). After 2 days, cells were treated with cytokines in GM for 5 days. In all experiments (excluded growth curve), 5000 SCs or FAPs were seeded in 96-multiwell plates (16,500 cells/cm^2^).

To perturb SCs proliferation process with muscle-derived EVs, after 2 days in sGM cells were switched in DMEM supplemented with 20% exosome-depleted heat-inactivated FBS (Gibco™, A2720803), 100 U/mL penicillin–streptomycin, 1mM sodium pyruvate, 10mM HEPES, and 4000 EVs/µL. Instead, to perturb differentiation process with muscle-derived EVs, after 3 days in sGM, SCs were switched in DMEM supplemented with 2% exosome-depleted heat-inactivated FBS (Gibco™, A2720803), 100 U/mL penicillin–streptomycin, 1mM sodium pyruvate, 10mM HEPES, and 4000 EVs/µL. In both assays, after 24h of treatment cells were fixed for immunofluorescence staining.

At the end of all experimental in vitro culture, cell plates were fixed to proceed with the immunofluorescence protocol described below.

### 2.5. Immunofluorescence

Cells were fixed with 2% paraformaldehyde solution for 20 min at room temperature (RT) and then washed 3 times with PBS. Permeabilization was performed with 0.5% Triton X-100 in PBS for 5 min at RT and washed 3 times with 0.1% Triton X-100 in PBS (washing solution—WS). Blocking was performed with 10% FBS in WS (blocking solution—BS) for 1 h at RT.

Immunolabeling was carried out in BS for 1 hour at RT with the following antibodies and dilutions: mouse anti-myogenin (1:250, Thermo Scientific, Cat. No. 14-5643-80), rabbit anti-PPARγ (1:200, Cell Signaling, Danvers, MA, United States, Cat. No. 2443S), and rabbit anti-Ki67 (1:400, Cell Signaling, Cat. No. 9129S).

After incubation with the primary antibodies, the specimens were washed 3 times with WS. Secondary antibodies were incubated for 30 min at RT in the dark using the following products and dilutions in BS: Goat anti-Mouse IgG (H+L) Cross-Adsorbed Secondary Antibody, Alexa Fluor 488 (Thermo Fisher Scientific, Waltham, MA, United States, Cat. No. A-11001, 1:300), Goat anti-Rabbit IgG (H+L) Cross-Adsorbed Secondary Antibody, Alexa Fluor 488 (Thermo Fisher Scientific, Cat. No. A-11008, 1:300).

After the incubation with the secondary antibodies the specimens were washed 3 times with WS. Nuclear staining was performed with Hoechst 33,342 (Thermo Fisher Scientific, Cat. No. H3570) diluted 1:5000 in WS. The specimens were washed 3 times with PBS and maintained at 4 °C in PBS supplemented with 0.02% sodium azide.

### 2.6. Oil Red O Staining

The Oil red O solution (Sigma-Aldrich, Cat. No. O0625) was used for detection of lipid droplets in adipocytes. The stock solution (0.5% filtered solution of Oil red O in isopropanol) was dissolved in ddH2O in 3:2 ratio and filtered. The cells were incubated for 5 min at RT, followed by two washings with PBS and Hoechst 33,342 staining.

### 2.7. Image Acquisition and Analysis

All microscopy images were acquired with a Leica DMI-6000B fluorescent microscope. In all the experiments, 20× and 40× images were acquired with the Tile scan mode using a 5 × 5 matrix-layout after auto-focusing. Image counts and quantifications were performed using the ImageJ software through both macros and manual analysis. Briefly, to correct uneven illuminated background, a “Rolling Ball” algorithm was used if needed. Then, histogram function was used to optimize the signal intensity, with the end to facilitate the application of an appropriate threshold. The obtained binary image was used for the quantification: for the counts of nuclei and nuclear signals, size and circularity cut-off were used to exclude false positive signals. Lastly, results were showed as “Bare outlines” and merged with raw images to compare the counted particles with original signals. If needed, this resulting merge was manually corrected.

### 2.8. RNAseq

For 3′-end RNAseq, immune cells, endothelial cells, SCs, and FAPs were purified from wt mice 3 days after cardiotoxin injury. Total RNA was extracted using Trizol according to the manufacturer’s recommendations. Libraries were prepared from 100 ng of total RNA using the QuantSeq 3′ mRNA-Seq Library Prep Kit FWD for Illumina (Lexogen GmbH, Vienna, Austria). The library quality was assessed by using High Sensitivity DNA D1000 Screen Tape (Agilent Technologies, Santa Clara, CA, USA). Libraries were sequenced on a NextSeq 500 using a highoutput single-end, 75 cycles, v2 Kit (Illumina Inc., San Diego, CA, USA). Approximately 44 × 10^6^ reads were obtained for each sample. Sequence reads were trimmed using the Trim Galore software 16 to remove adapter sequences and low-quality end bases (Q < 20). Alignment was performed with STAR 17 on the reference provided by UCSC Genome Browser 18 for Mus musculus (UCSC Genome Build mm10). The expression levels of genes were determined with htseq-count 19 using the Gencode/Ensembl gene model.

### 2.9. Extracellular Vesicles Isolation Protocols

Ultracentrifugation protocol was used to extract muscle-derived EVs. Uninjured and injured muscles (tibialis anterior, quadriceps, and gastrocnemius) were dissected from hind limbs, cutting the two tendons, and then cultured ex-vivo in DMEM supplemented with 20% exosome-depleted heat-inactivated FBS (Gibco™ A2720803), 100 U/mL penicillin–streptomycin, 1mM sodium pyruvate, and 10mM HEPES. To facilitate the release of EVs and to increase metabolite uptake, dissected muscles were longitudinally divided: in more detail, we performed a single cut on tibialis anterior, 2 cuts on gastrocnemius, and 3 cuts on quadriceps, following the myofiber direction from a tendon to the other extremity, without cutting the whole height of the muscle. Thus, we obtained bi-lamellar, tri-lamellar, and tetra-lamellar muscles, respectively. After 24 h, we collected and centrifuged the culture media at 2000× *g* for 1 h. Next, samples were further centrifuged at 20,000× g for 1 h and supernatant was filtered sequentially through 0.45 µm and 0.22 µm filters tips. Lastly, culture media were centrifuged at 120,000× *g* for 16 h in order to pellet EVs. The obtained pellet was resuspended in PBS.

### 2.10. EVs Flow-Cytometry Analysis

Single-vesicles flow cytometric analysis was performed using a CytoFLEX S (Beckman Coulter, Brea, CA, USA). As recommended by the manufacturer, Megamix-Plus FSC reagent (BioCytex, Marseille, France), consisting of 100 nm, 300 nm, 500 nm, and 900 nm fluorescent beads, and Megamix-Plus SSC reagent, (BioCytex) consisting of 160 nm, 200 nm, 240 nm, and 500 nm fluorescent beads, were mixed in order to obtain the Gigamix solution, successively used to set the EV size ranges. Prior to each analysis, we used CytoFLEX Daily Quality Control (QC) to verify the optical alignment and the fluidics system integrity of CytoFLEX.

To avoid any swarm detection phenomenon, EV samples dissolved in PBS were analyzed by flow cytometry performing a serial dilutions assay. ddH_2_O was used to clean the fluidics system before every analysis, until we reached a background noise minor of 150 events/s.

With the end to label EVs, we prepared stock solutions of Syto RNASelect (Invitrogen, Carlsbad, CA, USA, Cat. No. S32703) and Calcein AM (Invitrogen, Cat. No. C34852) at 1 mM. Then, 100 µL of EVs samples were incubated with 1 µL of stock solutions, to obtain a working concentration of 10µM for both dyes. After 20 min, samples were diluted with 300 µL of filtered PBS in order to stop reactions, and immediately analyzed through flow cytometer.

### 2.11. EVs Western Blot Analysis

1.5 × 10^6^ EVs were lysed in RIPA buffer (50 mM Tris–HCl, pH 8.0, 150 mM NaCl, 12 mM deoxycholic acid, 0.5% Nonidet P-40) containing a cocktail of protease and phosphatase inhibitors (Sigma, St. Louis, MO, USA). Protein concentration was obtained by Lowry method [39]. EVs samples were resuspended in the loading buffer and boiled at 99 °C for 5 min. Half of the resulting EV lysate were loaded on 15% SDS–PAGE and then subjected to Western blotting analysis. Nitrocellulose membranes were stained with anti-CD63 (EXOAB-CD63A-1, System Biosciences, Palo Alto, CA, USA), anti-FLOT1 (EXOAB- FLOT1-1, System Biosciences, Palo Alto, CA, USA), anti-CD9 (EXOAB- CD9-1, System Biosciences, Palo Alto, CA, USA), anti-CD81 (EXOAB-CD83A-1, System Biosciences, Palo Alto, CA, USA), primary antibodies at 1:1000 dilution. Subsequently, membranes were incubated with the appropriate horseradish peroxidase-conjugated secondary antibodies, 1:20000 dilution. Immuno-reactive bands were visualized by a FluorChem FC3 System (Protein-Simple, San Jose, CA, USA) after membranes staining with Enhanced Chemiluminescence ECL Selected Western Blotting Detection Reagent (GE Healthcare, Pittsburgh, PA, USA). The relative intensity of the immune-reactive bands was performed using the ImageJ Analysis Software 1.50i (National Institutes of Health, Bethesda, MD, USA).

### 2.12. Interaction between EVs and FAPs or SCs

Freshly isolated SCs and FAPs were seeded in sGM and GM, respectively. After 2 days, cells were switched to sGM and GM with 20% of exosome-depleted heat-inactivated FBS and incubated with 8000 SytoRNASelect-labelled EVs/µL. Unbound dye in labelled EVs preparations were previously eliminated through Exosome Spin Columns (MW3000) (Invitrogen, Carlsbad, CA, USA, Cat. No. 4484449) following manufacturer’s instructions. After 1.5 h, cells were washed 3 times with sterile PBS and then used for immunofluorescence staining.

### 2.13. Transmission Electron Microscopy (TEM) Measurements

A drop of each EVs sample was deposited onto a 300-mesh copper grid for electron microscopy covered by thin amorphous carbon film (20 nm). TEM grids were placed on a teflon tape and samples were incubated for 20 min to obtain a high particle concentration on the grid. The excess of liquid was removed by touching the grid with filter paper. Before the samples were completely dried, 10 µL of 2% aqueous phosphotungstic acid solution (pH-adjusted to 7.3 using 1 N NaOH) was deposited on the grid and removed by filter paper after 2 min.

Measurements were carried out by means of a FEI TECNAI 12 G2 Twin (FEI Company, Hillsboro, OR, USA), operating at 120 kV and equipped with an electron energy filter (Gatan image filter, Pleasanton, CA, USA) and a slow-scan charge-coupled device camera (Gatan multiscan).

### 2.14. DLS Measurements

Dynamic Light Scattering (DLS) was used to measure the size of EVs samples. A NanoZetaSizer (Malvern) apparatus working in baskscattering configuration (173°) and equipped with a 5 mW HeNe laser was employed. For each sample, three measurements have been performed, each measurement being the average of at least 15 sub-runs. Temperature was fixed at 25 °C in the thermostated cell. Size distribution of the samples have been determined by NNLS analysis of DLS correlation functions within the framework of Mie. To get number distribution, we considered the vesicle model implemented in the Malvern NanoZS Research Software, with a refractive index equal to 1.4, as reported in the literature for different large vesicles [40,41].

### 2.15. Statistical Analysis

Three independent biological replicates were performed for each experiment. Errors are presented as standard deviation (SD). Statistical significance was assessed using Student’s t-test or One-Way ANOVA according to the data set. Differences were considered statistically significant when *p*-value < 0.05.

## 3. Results

### 3.1. Kinetic of Cytokine Synthesis following Damage

We first aimed to characterize the soluble proteins secreted in vivo during muscle regeneration. To this end, we isolated a heterogeneous mixture of muscle mononucleated cells and we monitored the accumulation of proteins destined to be secreted at four time points (1, 3, 5, and 12 days, termed 1d, 3d, 5d, and 12d, respectively) after damage with cardiotoxin (CTX).

**Figure 1:**
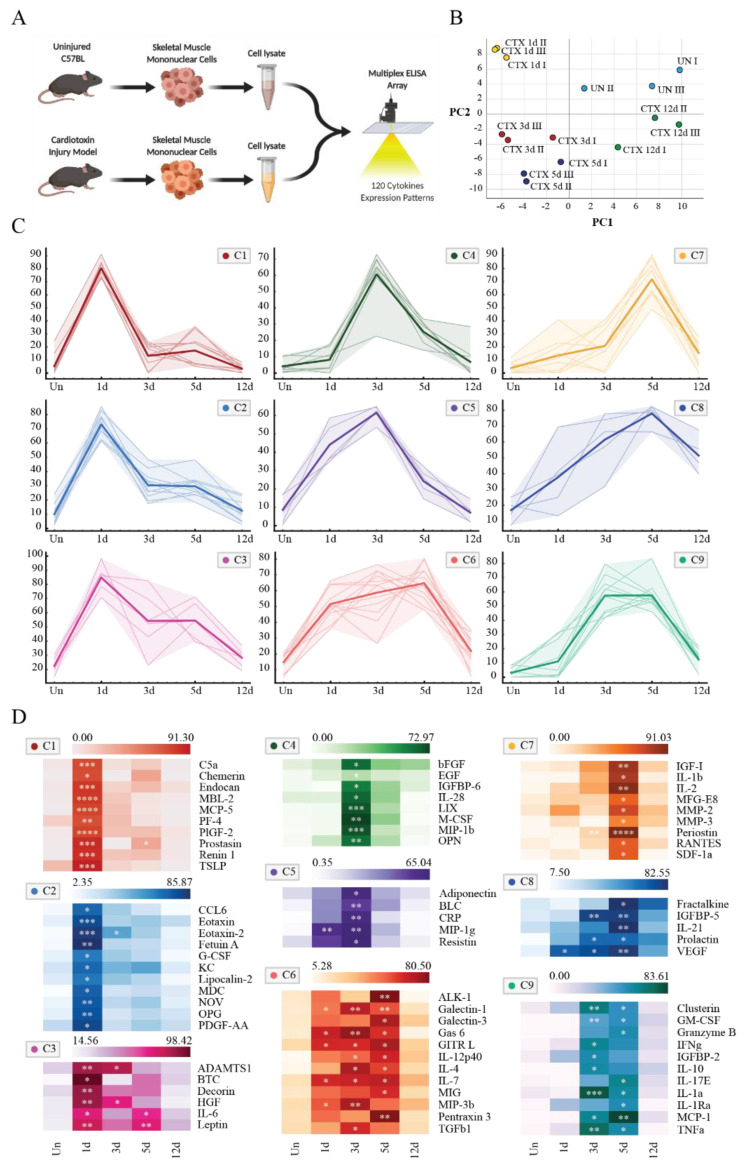
Analysis of cytokine expression during muscle regeneration. (**A**) The diagram illustrates the experimental procedure. Mononuclear cell fractions from uninjured muscles (UN) and at four different times after damage (CTX 1d, CTX 3d, CTX 5d, and CTX 12d) were isolated and lysed to determine the abundance of 120 cytokines by a multiplex ELISA array approach. Created with BioRender.com (**B**) PCA analysis using the entire dataset. Each dot represents a single replicate of three independent experiments. (**C**) Secreted protein expression patterns were clustered using a k-mean algorithm. In each graph the thin lines represent the single protein patterns while the thick one represents the average of all the expression patterns within the cluster. The shaded area indicates the variance within the cluster. (**D**) Heat maps of clusters illustrating the variation in concentration of all cytokines modulated during muscle regeneration. Each row represents a cytokine while the five columns are, from left to right, the five conditions UN, CTX 1d, CTX 3d, CTX 5d, and CTX 12d. Statistical significance was assessed by a One-Way ANOVA test: * *p* < 0.05; ** *p* < 0.01; *** *p* < 0.001; **** *p* < 0.0001.

The mononuclear cell fractions at each time-point were lysed immediately after isolation to limit the effect of perturbations caused by the isolation procedure, and the intracellular amount of 120 proteins destined to be secreted were measured by a multiplex ELISA array (Figure 1A).

Each time point can be seen as a vector whose coordinates are the normalized levels of the 120 proteins that are monitored. Biological replicates cluster in the principal component analysis (PCA) space (Figure 1B). The uninjured samples (UN) and the ones after recovery (CTX 12d) are positioned close in the two-dimensional space while they are separated from the regenerating samples (CTX 1d, CTX 3d, CTX 5d) along the first component (PC1). The regenerating samples, on the other hand, were arranged in a time-dependent order along the second component (PC2).

Of the 120 soluble proteins that we monitored in this assay, 76 were upregulated in response to cardiotoxin injury. 56 of these 76 secreted proteins are cytokines, 14 are soluble proteins playing a role in the inflammatory response, and 6 are proteases. The modulated proteins display characteristic kinetic trends raising in concentration after damage and reaching the maximum at different times. In order to compare the expression pattern of the 76 secreted proteins, we first normalized the data by setting the maximum observed value to 100 for each protein. We next applied a k-means clustering algorithm to group the secreted protein expression patterns according to their kinetic profile. We obtained nine clusters (C1 to C9) some of which are similar (i.e., clusters C1, C2, and C3) (Figure 1C). In Figure 1, D the expression data in each cluster are reported as heat maps detailing the expression of each protein and the corresponding statistical significance.

In conclusion, different secreted proteins are modulated during muscle regeneration, defining clear distinct time-dependent secretome profiles.

### 3.2. Inference of a Cell–Cytokine Interaction Network

**Figure 2:**
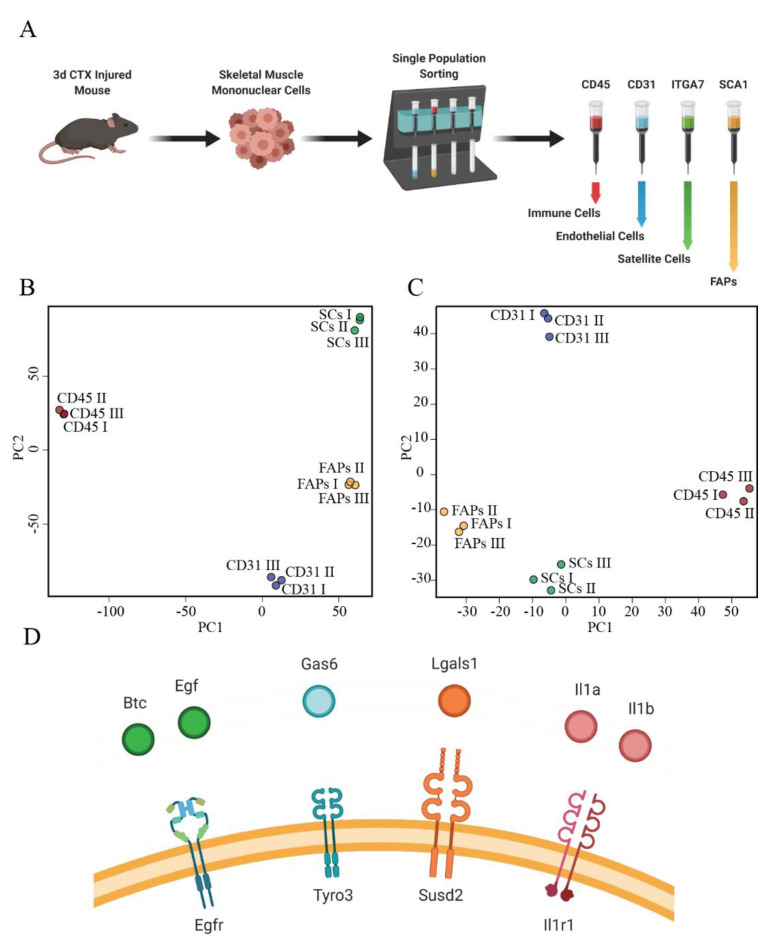
Inferred cytokine–receptor interactions in FAPs. (**A**) Representation of the experimental workflow. Three days after cardiotoxin injury, muscle–resident cell populations were sorted using MACS technology. The obtained cell fractions were immediately used to purify RNA. Created with BioRender.com (**B**) PCA analysis using the entire dataset and (**C**) only the mRNA coding secreted proteins. Each dot represents a replica of three independent experiments. (**D**) Schematic representation of the inferred cytokine–receptor pair resulting from the analysis of Multiplex ELISA and RNAseq data. Only receptors upregulated in FAPs, in comparison with the other cell types, are shown.

To gain insights into the cell–cell cross-talk that is modulated during the regeneration process, we characterized the expression levels of the cytokines and their receptors in the different cell types by RNAseq. For this analysis we focused on the third day after damage. This is a critical time-point as SCs are already fully activated to start the differentiation process, while, at the same time, the immune response switches from the pro-inflammatory to the anti-inflammatory phase. We determined the transcriptome of four mononuclear cell populations playing critical roles in muscle regeneration. We made use of antibodies conjugated to magnetic beads (Figure 2A) to sort sequentially (i) cells of the immune system as CD45+ cells, (ii) endothelial cells as CD45-/CD31+, (iii) SCs as CD45-/CD31-/α7int+ cells and iv) FAPs as CD45-/CD31-/α7int-/SCA1+ [42].

In a principal component analysis (PCA) (Figure 2B), the three biological replicates of each sample clustered according to cell type. A similar analysis carried out, using only the mRNA abundance of genes encoding secreted proteins [43], allowed the conclusion that the expression profiles of the mRNA for secreted proteins are significantly different in the four cell types (Figure 2C). This subset of genes for secreted proteins allowed us to characterize the predicted secretome in the four cell types (Supplementary Figure S1).

We next compared the list of secreted proteins that were found to be modulated in the multiplex ELISA analysis with those of the proteins predicted to be synthetized in the four cell types. For 55 of the 76 secreted proteins, we could identify at least one of the four cell types synthetizing the corresponding mRNA at day 3. Then, we aimed at inferring the cell types that, by expressing the matching receptors, could potentially sense these signals. To this end, we first used literature information to curate a table that associates each cytokine modulated in muscle regeneration to the corresponding receptor (Supplementary Table S1). The integration of this annotated table of cytokine–receptor pairs with the expression profiles of the four cell types allowed us to draw a network of potential cell–cell interactions at the third day of muscle regeneration (Supplementary Figure S2).

To validate our approach, we focused on the signals that FAPs can potentially sense and respond to. To this end we highlighted the interactions that involve a receptor upregulated in FAPs, in comparison with the other three cell populations, excluding those that involve cytokines not detected in both multiplex ELISA and RNAseq experiments. We noticed that the cytokines BTC, EGF, Gas6, Lgals1 (Galectin1), IL1α, and IL1β have their corresponding receptors expressed by FAPs (Figure 2D; Supplementary Figure S3) and we focused on these for further experimental validations.

### 3.3. Effect of Cytokines on FAP Differentiation

To gain insight on the potential functional effect of the seven cytokines inferred by our approach to target FAPs, we set out to treat purified FAPs with the cytokines identified by the analysis of the RNAseq and Multiplex ELISA data. We added to this list six cytokines: IL36g, as it is a member of the IL1 cytokine family and its receptor gene (Il1rl2) is transcribed in FAPs, IFNγ, and IL33 because the mRNAs of their corresponding receptors (Ifngr1 and Ifngr2 for IFNγ and Il1rl1 for IL33) are highly expressed in FAPs. Lastly, we also selected IL3, OPN (Osteopontin), and POSTN (Periostin) based on both RNAseq results and literature data [44,45]. Three days after acute injury we purified and treated FAPs with each of the 12 cytokines and monitored their potential to modulate FAP adipogenesis and fibrogenesis in vitro.

**Figure 3:**
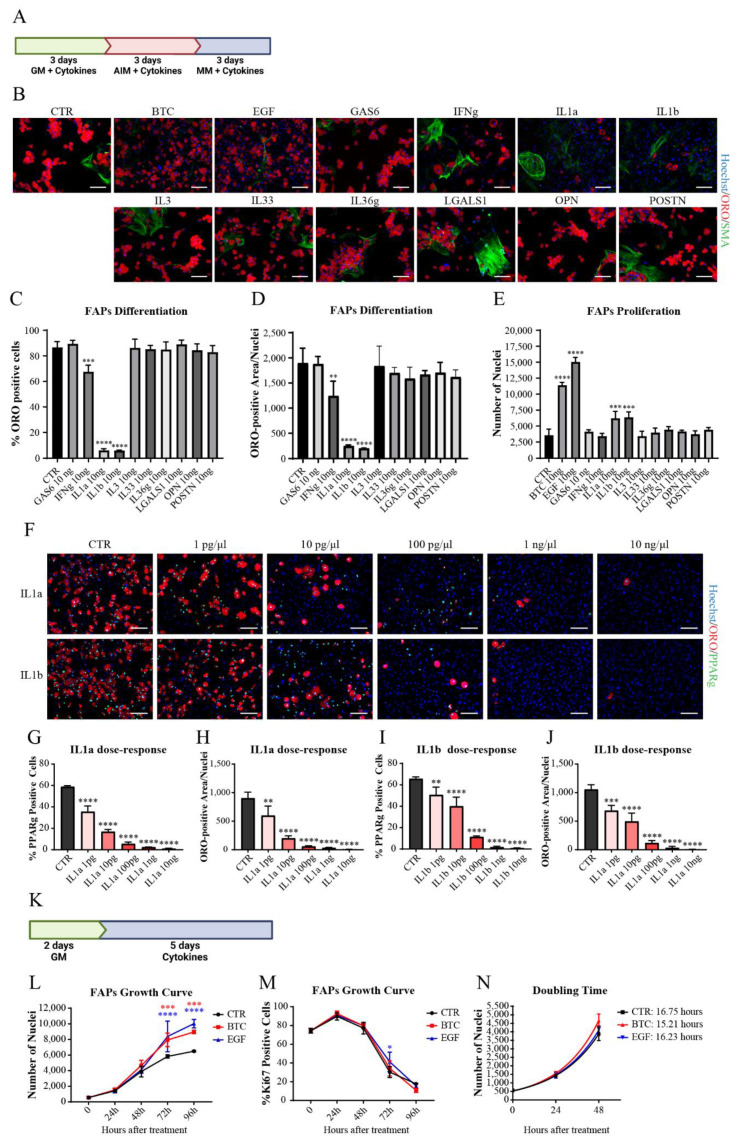
Modulation of FAPs adipogenesis and proliferation. (**A**–**E**) FAPs isolated 3 days after injury with cardiotoxin were plated and treated with 12 cytokines. (**A**) Experimental design of cell treatment. GM = Growth Medium. AIM = Adipogenic Induction Medium. MM = Maintenance Medium. Created with BioRender.com (**B**) Representative images showing anti-SMA immunofluorescence (green), ORO-staining (red) and nuclei staining with Hoechst 33,342 (blue). Scale Bar = 100 μm. (**C,D**) Quantification of adipogenic differentiation, represented as percentage of ORO-positive cells and ORO-positive area. (**E**) Nuclei count at the end of adipogenic differentiation. (**F**–**J**) FAPs isolated from 3 days injured mice were plated and treated with 1 pg, 10 pg, 100 pg, 1 ng, and 10 ng of IL1α or IL1β. (**E**) Representative anti-PPARγ immunofluorescence (green), ORO-staining (red), and nuclei staining with Hoechst 33,342 (blue). Scale Bar = 100 μm. (**F**–**I**) Quantification of adipogenic differentiation, represented as percentage of PPARγ positive nuclei and ORO-positive area, after treatment with IL1α and IL1β. (**K**) Experimental design of cell treatment. GM = Growth Medium. Created with BioRender.com (**L**–**N**) FAPs isolated from 3 days after cardiotoxin injury were plated at low confluence and treated with EGF and BTC to obtain a growth curve. (**L**) Nuclei count of both untreated FAPs (CTR) and treated with EGF and BTC. (**M**) Percentage of positive Ki67 nuclei at each time point. (**N**) Doubling time analysis of data in panel (**J**) with non-linear regression and Exponential growth equation tool of Graph Pad Prism. Data are presented as means ± SD of three independent experiments. Statistical analysis was performed by One-Way Anova test: * *p* < 0.5; ** *p* < 0.01; *** *p* < 0.001; **** *p* < 0.0001.

To perturb adipogenic differentiation, cells were cultured for 3 days in growth medium (GM), followed by 3 days in adipogenic induction medium (AIM) and finally 3 days in Maintenance Medium (MM) (Figure 3A); 10 ng/mL of each cytokine were added, starting from the very beginning, and were replenished with fresh cytokine every 3 days. To evaluate the fraction of differentiated cells in each condition, we stained with Oil Red O (ORO) to mark lipid droplets in order to quantify adipocytic differentiation and smooth muscle actin (SMA)-staining to visualize striking differences in myofibroblasts formation.

The majority of cytokines did not show any effect on adipogenesis and myofibroblast differentiation (Figure 3B). IL1α and IL1β, however, significantly and markedly inhibited the formation of adipocytes as confirmed both from adipocyte count (Figure 3C) and quantification of ORO-positive area (Figure 3D). IFNγ also had a significant negative, albeit less important, effect on FAP adipogenesis. EGF and BTC were able to promote proliferation of FAPs, consistently increasing the total number of nuclei (Figure 3E); because of the high cell density, for these two cytokines it was not possible to quantify the number of adipocytes. We next performed a dose–response analysis of the anti-adipogenic effect of IL1α and IL1β by testing different cytokine concentrations (Figure 3F). We confirmed that both IL1α and IL1β inhibit FAP adipogenesis with an IC50 of approximately 2 and 16 pg/mL, respectively (Figure 3G–J). Moreover, by drawing a growth curve (Figure 3K), we verified that both EGF and BTC promote the proliferation of FAPs (Figure 3L–N).

**Figure 4:**
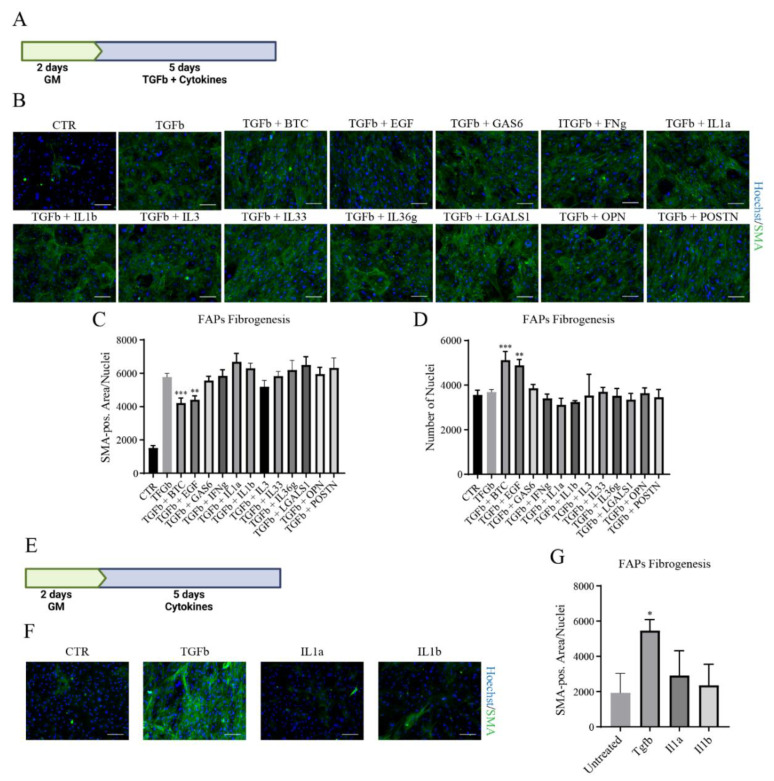
Modulation of FAPs Fibrogenesis. (**A**–**E**) FAPs isolated 3 days after injury with cardiotoxin were plated and treated with 12 cytokines to influence TGFb-induced fibrogenesis. (**A**) Experimental design of cell treatment. GM = Growth Medium. Created with BioRender.com (**A**) Representative images showing anti-SMA immunofluorescence (green) and nuclei staining with Hoechst 33,342 (blue). Scale Bar = 100 μm. (**B**,**C**) Quantification of fibrogenic differentiation, represented as SMA-positive area (pixel^2^). (**D**) Nuclei count at the end of in vitro differentiation. (**E**–**G**) FAPs isolated from 3 days injured mice were plated and treated with TGFβ or IL1α and IL1β. (**E**) Experimental design of cell treatment. GM = Growth Medium. Created with BioRender.com F) Representative anti-SMA immunofluorescence (green) and nuclei staining with Hoechst 33,342 (blue). Scale Bar = 100 μm. (**G**) Quantification of fibrogenic differentiation, represented SMA-positive area (pixel^2^). Statistical analysis was performed by One-Way Anova test: * *p* < 0.5; ** *p* < 0.01; *** *p* < 0.001.

We next tested the potential of the selected cytokines to interfere with fibrogenesis induced by TGFb. To this end, FAPs isolated 3 days after cardiotoxin injury were plated for 2 days in GM and then followed by incubation for 5 days with 1 ng/mL of TGFb and 10 ng/mL of each cytokine (Figure 4A). Cells were finally fixed and αSMA expression was evaluated to quantify fibrogenic differentiation. No cytokines inhibited fibrogenic differentiation induced by TGFb (Figure 4B,C). BTC and EGF quantifications show a reduction in αSMA-positive area, but it is probably a consequence of the higher nuclei density in the plate (Figure 4D). Lastly, since IL1a and IL1b, similarly to TGFb, are strong inhibitors of adipogenesis, we asked if they were also able to stimulate fibrogenesis. After 2 days in GM, we treated cells for 5 days with TGFb, IL1a, or IL1b (Figure 4E). Unlike TGFb, cells treated with IL1a and IL1b show an αSMA-positive area comparable to controls (F,G). In conclusion, 5 out of the 12 tested cytokines were shown to modulate either differentiation or proliferation. Interestingly, IL1a and IL1b inhibit in vitro adipogenic differentiation without promoting myofibroblast formation.

### 3.4. Characterization of Extracellular Vesiscles following Damage

Cell cross-talk can be mediated by secreted cytokines or by extracellular vesicles (EVs). Thus, we set out to characterize EVs released within regenerating muscles. Purifying EVs secreted in vivo by a solid tissue is challenging. In order to minimize the perturbation caused by the separation of the tissue from its natural context, we decided to extract EVs from muscles cultured in vitro. By this approach, muscle mononuclear cells and myofibers would still sense physiological stimuli from the tissue environment.

After 24 h of ex vivo culture, the culture media were collected and processed to isolate EVs smaller than 240 nm. This size-cut removes cellular debris and organelles that may derive from cells disrupted because of the procedure.

**Figure 5:**
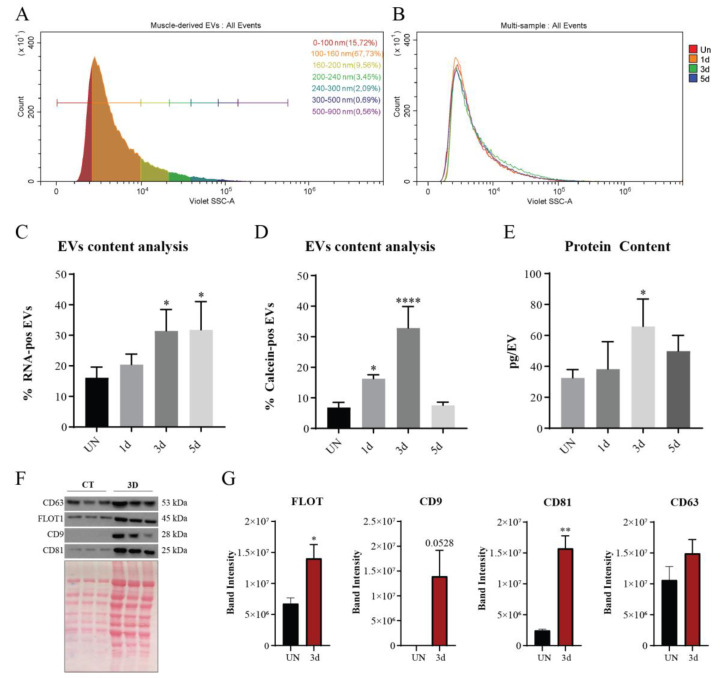
Muscle-derived EVs characterization. (**A**) Size distribution analysis of muscle-derived EVs. Size ranges 0–100 nm, 100–160 nm, 160–200 nm, 200–240 nm, 240–300 nm, 300–500 nm, and 500–900 nm are based on Gigamix fluorescent beads. (**B**) Overlay of UN, 1d, 3d, and 5d EVs size distributions. For each sample 100,000 EVs are displayed. (**C**) Quantitation of EVs labelled with Syto RNASelect and (**D**) calcein, represented as percentage of positive EVs. (**E**) Protein quantification of the EVs sample with the Lowry method normalized over the number of lysed EVs. (**F**) Representative western blot of proteins extracted from UN and 3d EVs. Ponceau staining was used to show the protein loading. (**G**) Densitometric quantification of bands in panel F. Data are presented as means ± SD of three independent experiments. Statistical analysis was performed by a One-Way Anova test. * *p* < 0.05; ** *p* < 0.01; **** *p* < 0.000.

Particle size was monitored by analyzing their scattering in the flow cytometer. The Violet SSC parameter led us to detect vesicles smaller than 100 nm. In all preparations, approximately 95% of the EVs had a diameter of 0–200 nm (Figure 5A), with hardly any difference among the profiles of the various conditions (Figure 5B; Supplementary Figure S4D). This result was confirmed by DLS and TEM analysis (Supplementary Figure S4A–C).

Next, we considered the possibility that macromolecule loading into the vesicles would be modulated along the regeneration process. To verify this hypothesis, we labeled EVs with SytoRNASelect and calcein in order to evaluate RNA and esterase content, respectively. RNA and calcein labelling revealed marked differences between the samples processed at different times during regeneration (Supplementary Figure S4E). The fraction of RNA-stained EVs increased monotonically (Figure 5C) while the percentage of calcein-positive EVs had a peak on the third day after acute damage (Figure 5D).

Calcein is a small molecule hydrolyzed by esterases. The ability to metabolize calcein reflects the quantity of uploaded esterases into their lumen. We considered that other proteins, similar to esterases, might be differently loaded into the vesicles. Thus, we also measured protein content in the vesicle preparations by the Lowry method. We found that protein content reached maximum by the third day of muscle regeneration and decreased by the fifth day, showing the same trend as the reactivity of EVs to calcein.

Finally, we analyzed by western blot the tetraspanin content in a comparable number of vesicles produced by dissected muscles. Tetraspanins are proteins involved in protein uptake into the vesicle lumen during EV biogenesis [46]. We focused on the third day of the regeneration process and confirmed by ponceau staining that vesicles in the 3d preparation contain a higher amount of proteins (Figure 5F). However, the 3 tetraspanins CD9, CD63, and CD81 are differently modulated in the 3d when compared to the uninjured samples (Figure 5G). CD9 is hardly detectable in the EV preparation from the uninjured muscle while it is overexpressed in the 3d samples. The expression of CD63 is comparable in the 2 EVs preparations while CD81 is significantly upregulated in the EVs derived from injured muscles. We also analyzed the expression of FLOT1, another protein which is involved in vesicle cargo upload [47] and we observed a significant overexpression in the 3d samples. The increase of tetraspanin expression, mainly CD9 and CD81, lends further support to the conclusion that the loading of the protein cargo into the vesicles is modulated during regeneration.

### 3.5. Interaction between EVs and Muscle–Resident Cell Populations

We next investigated the interaction of EVs with satellite cells and fibro/adipogenic progenitors, the two cell populations that play a major role in muscle regeneration [22].

**Figure 6:**
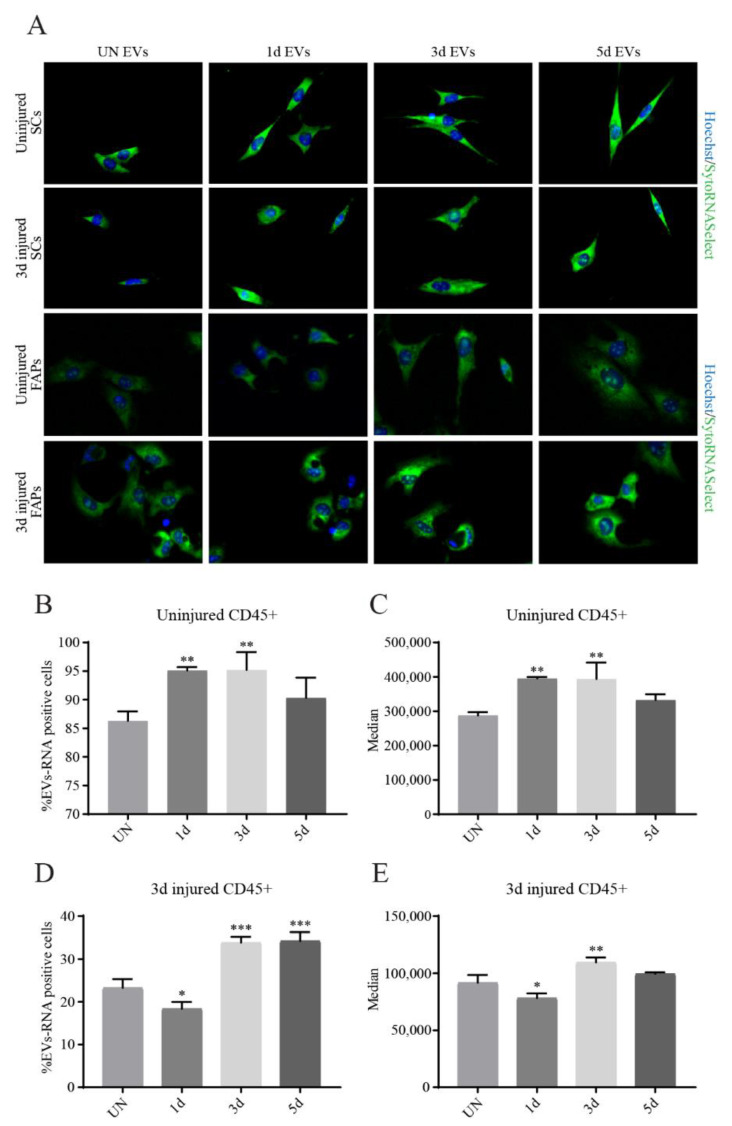
Interaction between EVs and mononuclear muscle cell populations. (**A**) Representative immunofluorescence of SCs and FAPs incubated with labelled EVs. Syto RNASelect signal and nuclei staining with Hoechst 33,342 are shown, respectively, in green and blue. (**B**) Percentage of CD45+ cells derived from uninjured muscles and (**C**) Signal intensity in cells incubated with EVs previously labelled with Syto RNASelect. (**D**) Percentage of CD45+ cells derived from 3d injured muscles and (**E**) Signal intensity in cells incubated with EVs previously labelled with Syto RNASelect. Data are presented as means ± SD of three independent experiments. Statistical analysis was performed by a One-Way Anova test. * *p* < 0.05; ** *p* < 0.01; *** *p* < 0.001.

To this aim, we used SytoRNASelect to stain RNA contained in EVs. The stained vesicles were incubated for 1.5 h with purified SCs or FAPs derived from uninjured and injured muscles after 3 days. The incorporation of fluorescent ribonucleic acids into the mononuclear cells was monitored by fluorescence microscopy. We used RNA instead of calcein staining because the excess of unconjugated label could be washed out using elution filters, thus avoiding possible artefacts. SCs and FAPs were cultured 2 days before incubation with labelled EVs, in order to allow cells to adhere to the plate. Both SCs and FAPs were incubated with EVs produced either from injured or from uninjured muscles (Figure 6A). However, no significant differences between the different EVs preparations were observed. Fluorescent signals could be observed in all the cells analyzed.

We also investigated the interaction between the muscle-derived immune cells and the EVs; to this end, a suspension of freshly isolated CD45+ cells derived from uninjured and 3d injured mice was treated with the labelled vesicle preparation. Fluorescence incorporation into the cell population was monitored by flow cytometry. A very high percentage of immune cells derived from uninjured muscles interacted with all the EV samples, reaching approximately 95% of positivity with 1d and 3d vesicles (Figure 6B,C). Instead, the percentage of positive cells (Figure 6D) and the signal median (Figure 6E) of immune cells derived from 3d injured muscles is significantly higher with EVs coming from 3d and 5d time-points, while the interaction is significantly lower with EVs purified from 1d injured muscles.

In conclusion, the EVs samples were uptaken by the three cell populations that we have tested.

### 3.6. Effect of EVs on FAPs and SCs Proliferation and Differentiation

**Figure 7:**
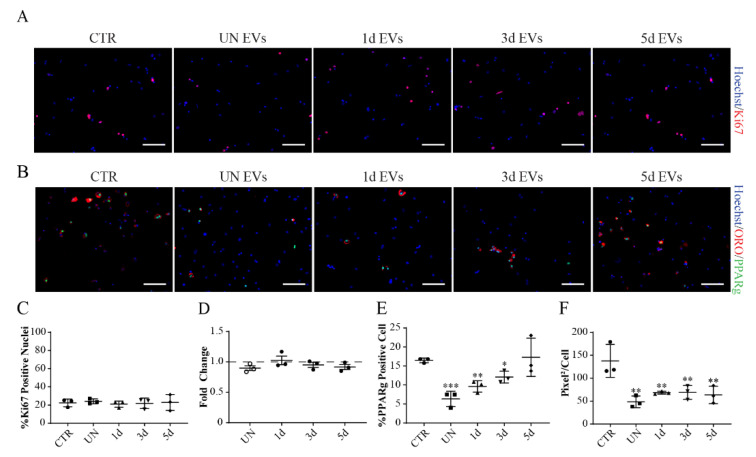
Muscle-derived EVs affect FAPs differentiation. (**A**,**C**,**D**). FAPs isolated from 3d injured muscles were plated and treated with EVs for 72 h. (**A**) Representative anti-Ki67 immunofluorescence (red) and nuclei staining with Hoechst 33,342 (blue). Scale Bar = 100 μm. (**C**) Quantification of Ki67 expression in FAPs treated with vesicles. (**D**) Nuclei count represented as Fold Change compared to the untreated control. (**B**,**E**,**F**) FAPs isolated from 3d injured muscles were plated induced to differentiate and treated with EVs. (**D**) Representative anti-PPARγ immunofluorescence (green), ORO-staining (red) and nuclei staining with Hoechst 33,342 (blue). Scale Bar = 100 μm. (**E**) Percentage of PPARγ-positive nuclei. (**F**) Ratio between the total stained area with ORO and nuclei number. Data are presented as means ± SD of three independent experiments. Statistical analysis was performed by a One-Way Anova test: * *p* < 0.05; ** *p* < 0.01; *** *p* < 0.001.

Next, we asked whether any of the EV preparations (UN, 1d, 3d, and 5d samples) had a positive or negative effect on FAP or SC proliferation and differentiation. We first looked at FAP proliferation by treating purified FAPs from injured muscles after 3 days of cardiotoxin injection for 72 h in vitro. No vesicle preparation affected FAP proliferation (Figure 7A), as assessed either by monitoring the percentage of Ki67-positive cells (Figure 6 and Figure 7) or the nuclei count (Figure 6 and Figure 7).

We next looked at the effect of EVs on FAP adipogenic differentiation by treating the same purified cells along the in vitro differentiation protocol, consisting of three days in Growth Medium (GM) and three days in Adipogenic Induction Medium (AIM). As assessed by the quantification of PPARγ-positive nuclei, the vesicles produced by the uninjured muscles show the largest inhibition of FAP adipogenic commitment. The effect is less prominent when using vesicles purified from the supernatant of muscles in a later stage of regeneration (Figure 7B,E,F). Nonetheless, FAPs treated with all the vesicle preparations showed the same decrease of the total area of lipid droplets (Figure 7F), suggesting a negative influence on terminal adipogenic differentiation.

In addition, we asked if the purified vesicles could carry molecular messages that modulate the proliferation and differentiation of satellite cells derived from uninjured muscles. We noticed that satellite cells incubated with some EV preparations were able to increase both the number of nuclei and the number of in vitro myotubes in comparison to untreated controls (data not shown). To exclude the possibility that promotion of the myogenic program resulting in a larger number of myotubes was a consequence of the increase in the proliferation rate that might influence differentiation, we treated the cells for a shorter time (24 h). To monitor the effect of vesicles on SC proliferation, we added EVs two days after plating, when cells are still in an active proliferative phase, while to monitor the effect on differentiation we treated cells 3 days after plating, when cells stop proliferating and start the commitment process.

**Figure 8:**
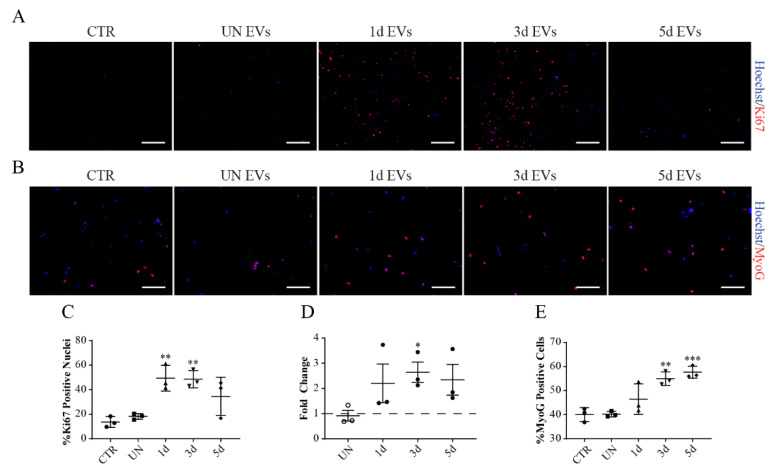
Muscle-derived EVs affect SCs proliferation and differentiation. (**A**,**C**,**D**) SCs isolated from uninjured muscles were plated for 2 days and then treated with EVs for 24 hours in GM. (**A**) Representative anti-Ki67 immunofluorescence (red) and nuclei staining with Hoechst 33,342 (blue). Scale Bar = 100 μm. (**C**) Quantification of Ki67 expression in SCs treated with vesicles. (**D**) Nuclei count represented as Fold Change compared to the untreated control. (**B**,**E**) SCs isolated from uninjured muscles were plated for 3 days and then treated for 24 h in DM. (**B**) Representative anti-MYOG immunofluorescence (red) and nuclei staining with Hoechst 33,342 (blue). Scale Bar = 100 μm. (**E**) Percentage of MYOG-positive nuclei. Data are presented as means ± SD of three independent experiments. Statistical analysis was performed by a One-Way Anova test: * *p* < 0.05; ** *p* < 0.01; *** *p* < 0.001.

The vesicles produced during the 1st and 3rd day after damage stimulated proliferation (Figure 8A), as assessed both by the fraction of Ki67-positive nuclei (Figure 8C) and nuclei count (Figure 8D). 5d EVs have some effect, yet not statistically significant.

As for differentiation, 3d and 5d EVs showed the greatest induction of terminal commitment (Figure 8B) as the percentage of MYOG-positive nuclei, which is a marker of late differentiation [48], increased in the 3d and 5d samples (Figure 8E). This increase of differentiation and SCs fusion after treatments with 3d EVs is in agreement with the increase of CD9 and CD81 [49].

In conclusion, vesicles derived from uninjured and injured muscles negatively affect FAP adipogenic differentiation, while having hardly any effect on cell proliferation. On the other hand, vesicles derived from injured muscles were able to stimulate SCs proliferation and differentiation. EVs produced in the early phases of muscle regeneration stimulate proliferation, while those produced in the late phases principally induce their differentiation.

## 4. Discussion

The fate of staminal cells in the muscle niche is modulated by the integration of a variety of signals from the muscle niche. Signals mediated by cytokines or EVs, substrate attachment, and cell–cell contacts determine the fate of the different cell populations [28] [50]. Here, we have investigated, by a variety of approaches, the changes in composition of the interstitial milieu during muscle regeneration, and we have looked into some of the functional consequences. We have characterized the kinetics of accumulation of 120 secreted proteins, and we have shown how the EVs content and properties vary during the regeneration process. Finally, we have delineated, by genome-wide RNAseq, the potential for four muscle cell populations to synthetize signaling molecules and for the receptors to process these signals. Collectively, these datasets represent a resource that can be freely used for further characterization and functional validation. Here, we have focused on the characterization of some cytokines that have the potential to control FAPs adipogenesis and on the ability of EVs to modulate FAPs and SCs proliferation and differentiation.

Monitoring extracellular signaling molecules in vivo on a large scale is beyond our current capabilities. Cell secretion is sensitive to signals coming from the microenvironment, and manipulation of donor cells can lead to artefacts. Indeed, changes in extracellular signals and substrate attachment are able to influence the cell secretion profile [51,52,53,54,55,56]. For these reasons, in our experimental design we have aimed at limiting perturbations on donor cells. To this end, we worked on biological samples whose conditions were, as far as possible, close to the physiological ones with cytokine and EV donor cells still adherent to their in vivo substrates. Hence, we isolated the mononuclear cell fraction from injured and uninjured muscles to analyze time dependent protein expression patterns for 120 secreted proteins, 76 of which were found to be modulated along the regenerative process. Similarly, as in vivo studies of EVs remain challenging [57], we had to rely on ex vivo culture to obtain EVs from a solid tissue. Muscle-related vesicles have been mostly studied by in vitro approaches by analyzing vesicles produced by a seeded single cell population [31,58]. In our work, we performed ex-vivo culture of dissected injured or uninjured muscles in order to maintain the physiological cell contacts and stimuli within the tissue. To our knowledge this is the first detailed characterization of muscle-derived EVs aimed at analyzing their content all along the regeneration process by a single-vesicles flow cytometry approach. Combining cytokines and EVs data, our work provides a description of the muscle secretome during regeneration.

EVs pose an additional challenge as quantitative data from different studies are often difficult to compare because of little agreement on normalization strategies. Noteworthy is the normalization used in our EVs experiments. In fact, standard protocols and procedures to facilitate result comparison and to improve experimental reproducibility have not been agreed upon [59]. Common normalizers are protein amounts or donor cell number. As equally expressed EV markers or intralumen proteins do not exist, we normalized our data on the number of EVs. This allowed us to conclude that the EVs protein cargo is significantly modulated during muscle regeneration.

Following damage, the complex cross-talk between leukocytes, mesenchymal stem cells, angiogenic cells, and muscle stem cells is essential for the recovery of tissue functionality [60]. The third day of muscle regeneration is a crucial time point, with the transition from the pro-inflammatory to the anti-inflammatory phase. This switch is driven by extracellular signals that also induce satellite cells terminal commitment [61]. In the present study, we have investigated the cell–cell interactions during the third day of muscle regeneration. By combining the results of a multiplex ELISA array approach, RNAseq experiments and in silico analyses, we obtained a picture of an inferred cell–cell interaction network at this time point. We focused on interactions mediated by receptors predominantly synthetized by fibro/adipogenic progenitors. This approach led us to identify IL1α and IL1β as potent inhibitors of FAP adipogenic differentiation. These two cytokines, which are produced by immune cells, are associated with the inflammatory response and have not been implicated in the regulation of mesenchymal cell differentiation in skeletal muscle yet [62]. Macrophages are already known to play a role in the regulation of FAP fate as in the final phases of muscle regeneration, they secrete TNFα that targets the expanded FAP population to undergo apoptosis, restoring the physiological concentration [63]. Moreover, macrophages polarized with IL1β secrete molecules are able to inhibit FAPs adipogenesis [64].

When cultivated in vitro, FAPs spontaneously differentiate into fibroblasts and adipocytes [16,18]. In the muscle niche, however, FAP differentiation is tightly controlled by anti-adipogenic signals. Different signals have been reported to have the potential to restrain FAP adipogenic differentiation in vitro [15,24,25,26,27,28,29,30,65,66]. Our results add a potential involvement of signals carried by EVs to limit adipogenesis in the homeostatic muscle. Toward the end of the regeneration, EVs gradually lose this anti-adipogenic potential, becoming ineffective in the late phases. Cytokines such as IL1α and IL1β rise in concentration in the regenerating muscle and may compensate for the decrease in the anti-adipogenic signal mediated by EVs. In muscular dystrophy, chronic inflammation is driven by pro-inflammatory cytokines such as TNFα, IL1α, and IL1β [67]. In this context, FAPs are the major source of infiltrating ectopic tissue [18]. The IL1α and IL1β signaling pathway should be further investigated in muscular dystrophies models. Over the past years, antagonists of the interleukin-1 receptor have been tested in a mouse model of muscular dystrophy with no favorable outcomes [68]. Our results, revealing the potential involvement of the interleukin 1 pathway in the control of FAP adipogenesis, are consistent with a protective role played by its activation and advise against therapeutic strategies based on its inhibition.

FAP depletion delays muscle regeneration after cardiotoxin-induced injury and causes a reduction of muscle stem cells proliferation, resulting in long-term atrophy [14]. Interestingly, this phenotype is similar to the one of the IL-1 KO mouse model, in which the absence of the two isoforms of the cytokine results in a compromised muscle regeneration due to the direct impairment of inflammatory response and satellite cell proliferation [62]. In this model it would be interesting to study the FAP transcriptome and compare it with the wild type one. As IL1 influences FAP differentiation, we should consider the hypothesis that FAPs, if not stimulated by IL-1, are not able to fully accomplish their role in muscle regeneration, possibly contributing to the IL-1 KO mouse phenotype.

Overall, our work provides a broad characterization of the muscle secretome and offers new insights into the complex in vivo cell–cell communication network during muscle regeneration.

## Data Availability

All data generated or analyzed in this study are available upon reasonable request.

## Supplementary Materials

The following are available online at https://www.mdpi.com/article/10.3390/biom11081171/s1, Figure S1: Related to Figure 2; Enrichment analysis of exclusive inferred secretome of single populations, Figure S2: Related to Figure 2; Inferred cell-cell interactions, Figure S3: Related to Figure 2; cytokines and corresponding receptor expression, Figure S4: Related to Figure 5; characterization of muscle-derived EVs, Figure S5: Related to Figure 5; original unedited blots, Table S1: Manually curated cytokine/receptor pairs and protein-protein interactions (PPI) involving secreted molecules.
