# Supplementary material for: Characterization of the Skeletal Muscle Secretome Reveals a Role for Extracellular Vesicles and IL1α/IL1β in Restricting Fibro/Adipogenic Progenitor Adipogenesis
Source: Biomolecules. 2021 Aug 8;11(8):1171. doi: 10.3390/biom11081171 (PMC8392554; doi:10.3390/biom11081171)
Supplement: Supplementary file 1 [file biomolecules-11-01171-s001.zip › biomolecules-1277182-supplementary.pdf]

Supplementary

Figure S1. *Related to Figure 2; Enrichment analysis of exclusive inferred secretome of single populations*

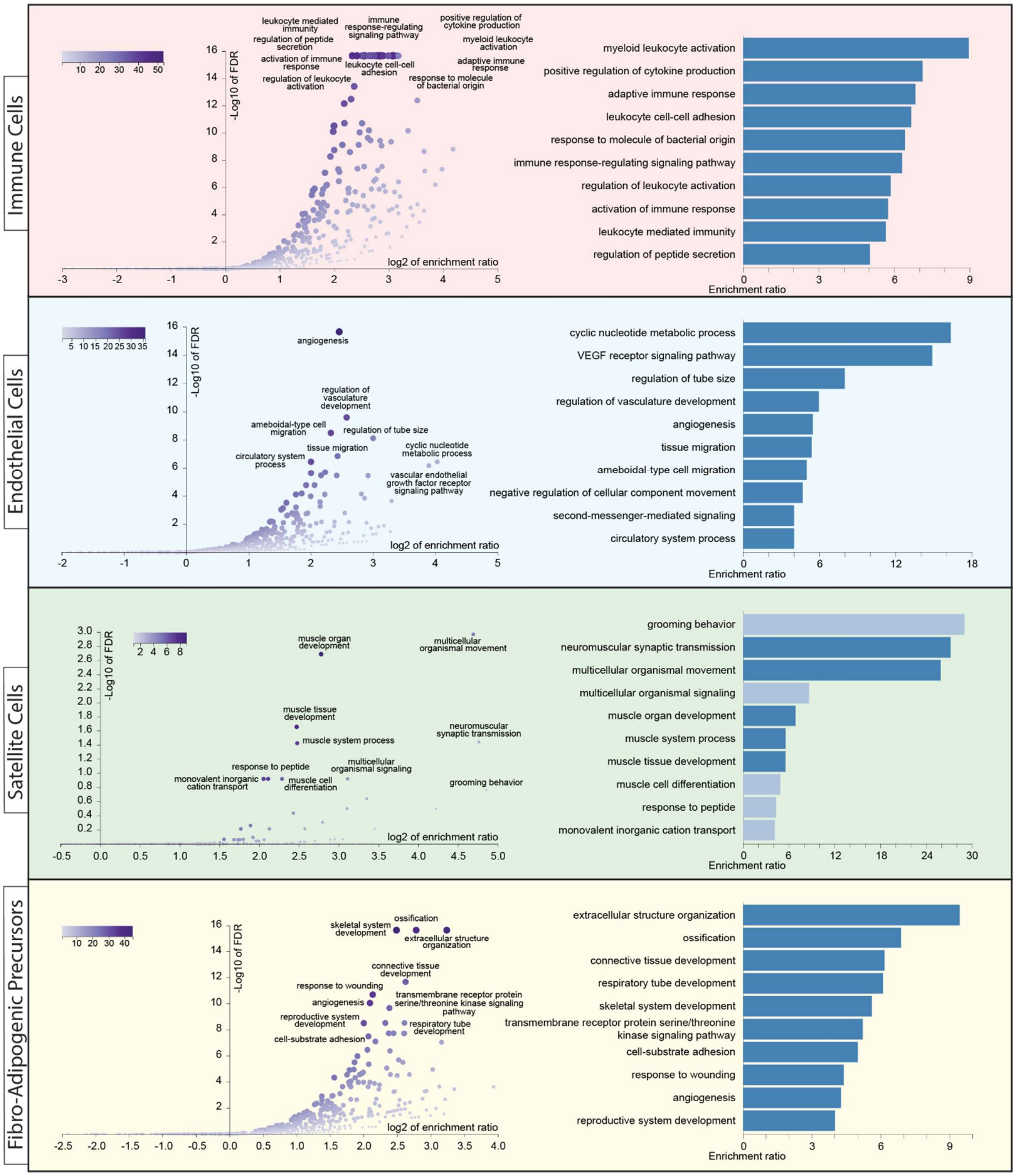

Analysis of the secretomes inferred from the transcriptome datasets offer hints about the biological processes the four different cell types may be involved in. We selected for each population only the mRNAs associated with secreted proteins that had a number of reads with a log2 Fold Change > 2 compared to the other three cell populations. Next, we used these lists of gene names to perform an enrichment analysis using the WEB-Gestalt tool (Meinken, Walker, Cooper, & Min, 2015). In ***Figure S1*** *the enriched Gene-Ontology Biological-Process terms are represented both as volcano and as bar plots. In the volcano plot (-Log10 of FDR vs Log2 of enrichment ratio), every dot represents a biological process. The biological processes that were found to be active, as judged from the expression of secreted proteins, were consistent with the described function of each cell population: i) the immune cells secreting proteins involved in the inflammatory and immune response; ii) the endothelial cells producing proteins that stimulate angiogenesis and cell migration; iii) satellite cells producing cytokines involved in the development and support of muscle differentiation; iv) fibro/adipogenic progenitors being predicted to coordinate a wide range of processes, including angiogenesis, development of extracellular structures and tissue modelling. In addition, FAPs were also predicted to produce a number of cytokines involved in the support of the skeletal system, suggesting a cross-talk between muscle and bones during muscle regeneration.*

Figure S2. *Related to Figure 2; Inferred cell-cell interactions.*

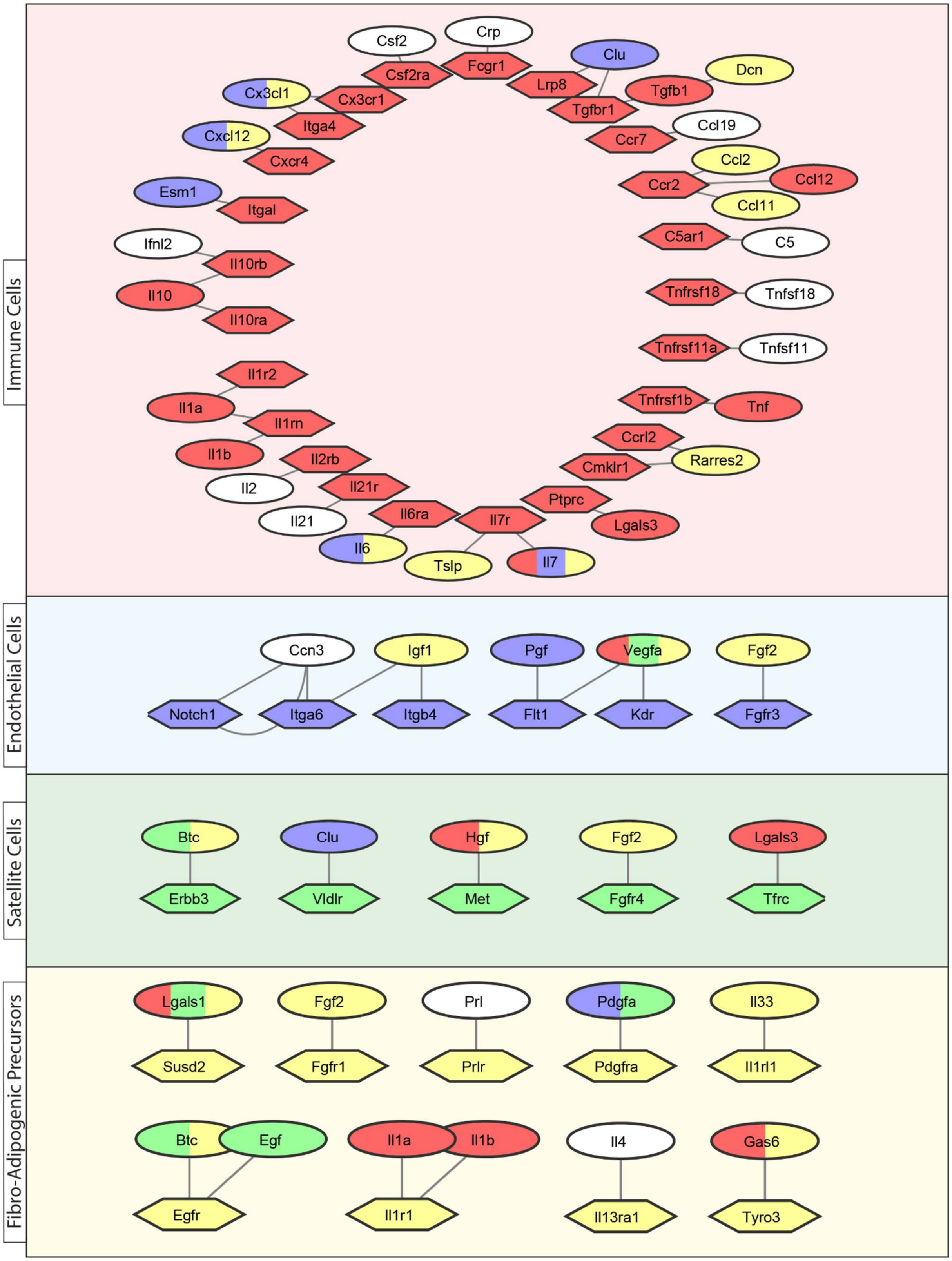

Scheme of inferred cell-cell interactions. Receptors and cytokines are represented as hexagons and ellipses respectively. Color code indicates which cell population overexpresses the corresponding mRNA with a log2 Fold Change > 2 compared to the other cell populations: red for immune cells, blue for endothelial cells, green for satellite cells and yellow for fibro-adipogenic progenitors. Ellipses filled with more than one colour indicate that the mRNAs of the corresponding cytokines are upregulated in more than one cell population. Cytokines whose mRNAs were not detected are represented as white ellipses. For each cell population, only the cytokine-receptor interactions that involve upregulated receptors are shown.

Figure S3. *Related to Figure 2; cytokines and corresponding receptor expression*

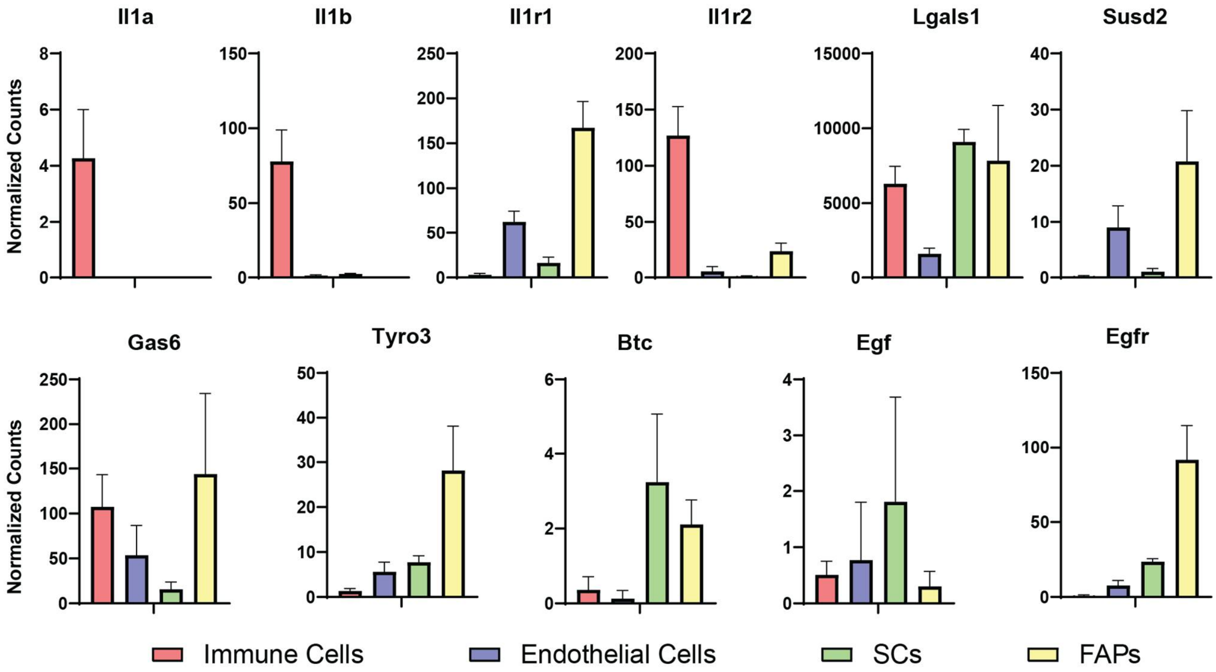

Normalized counts are shown for each cytokine and receptor selected to be investigated. The colour code indicates the cell type: red for immune cells, blue for endothelial cells, green for satellite cells and yellow for fibro-adipogenic progenitors. Data are presented as means ± SD of three independent experiments.

Figure S4. *Related to Figure 5; characterization of muscle-derived EVs.*

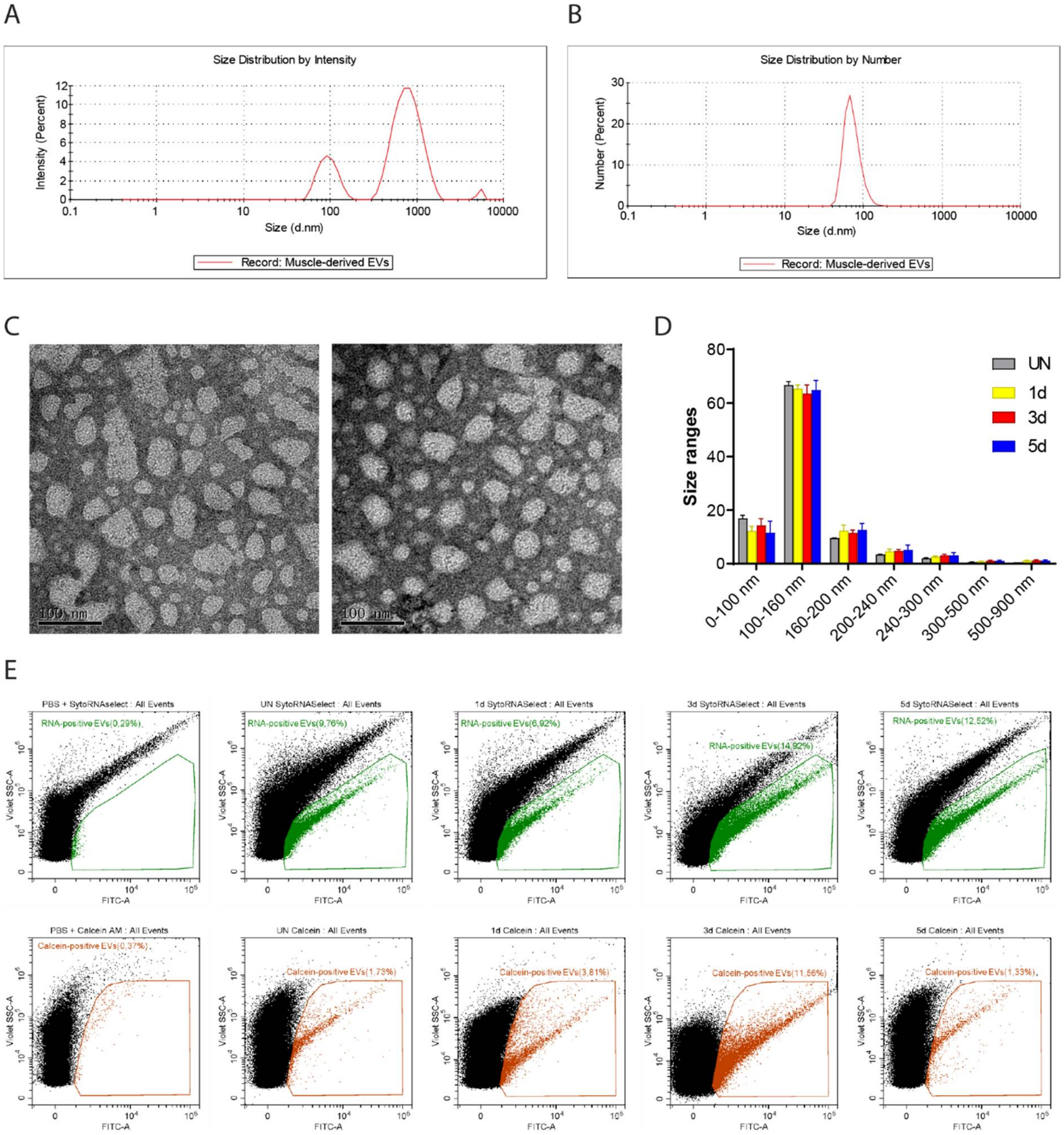

A-B) DLS analysis of muscle-derived EVs. A) Intensity-weighted and B) number-weighted size distributions hydrodynamic diameter of a representative muscle-derived EV sample. From the comparison of the two analysis method, it can be derived that the large majority of EVs have a size around 100 nm, while larger objects are practically absent. C) TEM images of negatively stained muscle-derived EVs after incubation with phosphotungstic acid, which confirm the presence of vesicles with size smaller than 100 nm. D) Size distribution of muscle-derived EV samples; there are no significant differences between the four preparations. Data are presented as means ± SD of three independent experiments. E) Dot-plot (Violet-SSC vs FITC-A) of labelled EVs with SytoRNASelect and Calcein. The events outside the gates are negative EVs and unbound dyes.

Figure S5. *Related to Figure 5; original unedited blots.*

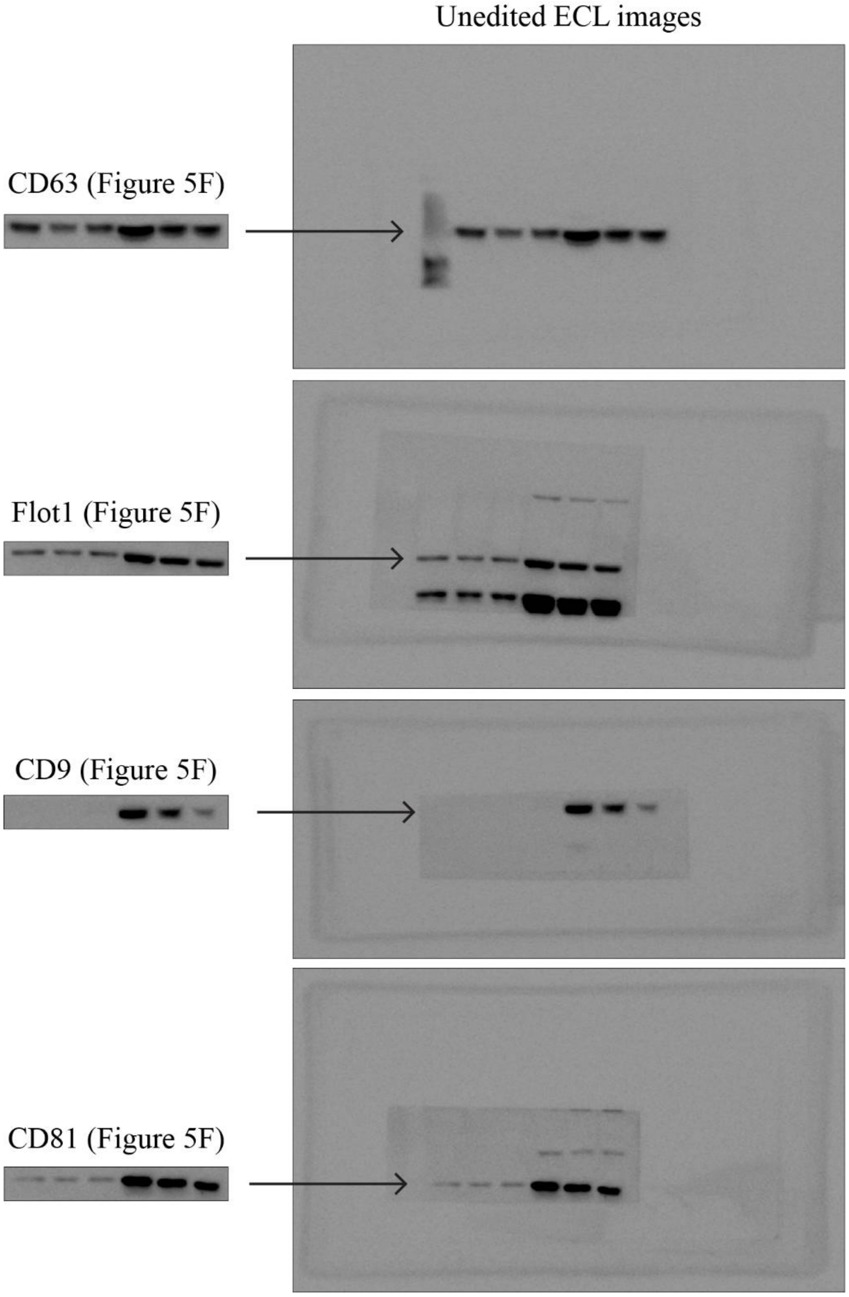

Supplementary Table 1: Manually curated cytokine/receptor pairs and protein-protein interactions (PPI) involving secreted molecules.

UNIPROT ID CYTOKINE	GENE NAME CYTOKINE	UNIPROT ID RECEPTOR	GENENAME RECEPTOR	TYPE OF INTERACTION
Q60994	Adipoq	Q91VH1	Adipor1	REC/CYT
Q60994	Adipoq	Q8BQS5	Adipor2	REC/CYT
P29699	Ahsg	Q9QUK6	Tlr4	REC/CYT
Q05928	Btc	Q01279	Egfr	REC/CYT
Q05928	Btc	P70424	Erbb2	REC/CYT
Q05928	Btc	Q61526	Erbb3	REC/CYT
P06684	C5	P30993	C5ar1	REC/CYT
P48298	Ccl11	P51678	Ccr3	REC/CYT
P48298	Ccl11	P51683	Ccr2	REC/CYT
P48298	Ccl11	P51680	Ccr4	REC/CYT
Q62401	Ccl12	P51683	Ccr2	REC/CYT
O70460	Ccl19	P47774	Ccr7	REC/CYT
O70460	Ccl19	Q924I3	Ackr4	REC/CYT
P10148	Ccl2	P51683	Ccr2	REC/CYT
O88430	Ccl22	P51680	Ccr4	REC/CYT
Q9JKC0	Ccl24	P51678	Ccr3	REC/CYT
P14097	Ccl4	P51682	Ccr5	REC/CYT
P30882	Ccl5	P51675	Ccr1	REC/CYT
P30882	Ccl5	P51678	Ccr3	REC/CYT
P30882	Ccl5	P51680	Ccr4	REC/CYT
P30882	Ccl5	P51682	Ccr5	REC/CYT
P30882	Ccl5	Q6X632	Gpr75	REC/CYT
P27784	Ccl6	P51675	Ccr1	REC/CYT
P51670	Ccl9	P51675	Ccr1	REC/CYT
Q64299	Ccn3	Q01705	Notch1	REC/CYT
Q64299	Ccn3	P43406	Itgav	REC/CYT
Q64299	Ccn3	P11688	Itga5	REC/CYT
Q64299	Ccn3	P09055	Itgb1	REC/CYT
Q64299	Ccn3	O54890	Itgb3	REC/CYT
Q06890	Clu	P98156	Vldlr	REC/CYT
Q06890	Clu	Q64729	Tgfbr1	REC/CYT
Q06890	Clu	Q62312	Tgfbr2	REC/CYT
Q06890	Clu	Q924X6	Lrp8	REC/CYT
P14847	Crp	P26151	Fcgr1	REC/CYT
P07141	Csf1	P09581	Csfr1	REC/CYT
P01587	Csf2	Q00941	Csf2ra	REC/CYT
P01587	Csf2	P26955	Csf2rb	REC/CYT
P09920	Csf3	P40223	Csf3r	REC/CYT
O35188	Cx3cl1	Q9Z0D9	Cx3cr1	REC/CYT

O35188	Cx3cl1	P43406	Itgav	REC/CYT
O35188	Cx3cl1	O54890	Itgb3	REC/CYT
O35188	Cx3cl1	Q00651	Itga4	REC/CYT
O35188	Cx3cl1	P09055	Itgb1	REC/CYT
P12850	Cxcl1	P35343	Cxcr2	REC/CYT
P40224	Cxcl12	P70658	Cxcr4	REC/CYT
P40224	Cxcl12	P56485	Cxcr7	REC/CYT
P40224	Cxcl12	O54890	Itgb3	REC/CYT
O55038	Cxcl13	Q04683	Cxcr5	REC/CYT
Q9WUQ5	Cxcl14	Unknown	Unknown	Unknown
P50228	Cxcl5	P35343	Cxcr2	REC/CYT
P18340	Cxcl9	O88410	Cxcr3	REC/CYT
P28654	Dcn	P04202	Tgfb1	Inhibition
Q9JI71	Dll4	Q01705	Notch1	REC/CYT
Q9JI71	Dll4	P31695	Notch4	REC/CYT
P01132	Egf	Q01279	Egfr	REC/CYT
Q9QYY7	Esm1	P24063	Itgal	REC/CYT
P15655	Fgf2	P16092	Fgfr1	REC/CYT
P15655	Fgf2	P21803	Fgfr2	REC/CYT
P15655	Fgf2	Q61851	Fgfr3	REC/CYT
P15655	Fgf2	Q03142	Fgfr4	REC/CYT
Q61592	Gas6	Q61592	Axl	REC/CYT
Q61592	Gas6	P55144	Tyro3	REC/CYT
Q61592	Gas6	Q60805	Mertk	REC/CYT
Q08048	Hgf	P16056	Met	REC/CYT
Q01279	Ifng	P15261	Ifngr1	REC/CYT
Q01279	Ifng	Q63953	Ifngr2	REC/CYT
Q4VK74	Ifnl2	Q61190	Il10rb	REC/CYT
Q4VK74	Ifnl2	Q8CGK5	Ifnlr1	REC/CYT
P05017	Igf1	Q60751	Igf1r	REC/CYT
P05017	Igf1	P43406	Itgav	REC/CYT
P05017	Igf1	O54890	Itgb3	REC/CYT
P05017	Igf1	Q61739	Itga6	REC/CYT
P05017	Igf1	A2A863	Itgb4	REC/CYT
P47877	Igfbp2	P05017	Igf1	PPI
P47877	Igfbp2	P09535	Igf2	PPI
Q07079	Igfbp5	P05017	Igf1	PPI
P47880	Igfbp6	P05017	Igf1	PPI
P47880	Igfbp6	P09535	Igf2	PPI
P18893	Il10	Q61727	Il10ra	REC/CYT
P18893	Il10	Q61190	Il10rb	REC/CYT
P43432	Il12b	Q60837	Il12rb1	REC/CYT
P43432	Il12b	Q8K4B4	Il23r	REC/CYT
P01582	Il1a	P13504	Il1r1	REC/CYT
P01582	Il1a	P27931	Il1r2	REC/CYT
P10749	Il1b	P13504	Il1r1	REC/CYT

P10749	Il1b	P43406	Itgav	REC/CYT
P10749	Il1b	P11688	Itga5	REC/CYT
P10749	Il1b	P09055	Itgb1	REC/CYT
P10749	Il1b	P25085	Il1rn	Inhibition
P01582	Il1a	P25085	Il1rn	Inhibition
P04351	Il2	P01590	Il2ra	REC/CYT
P04351	Il2	P16297	Il2rb	REC/CYT
P04351	Il2	P34902	Il2rg	REC/CYT
Q9ES17	Il21	Q9JHX3	Il21r	REC/CYT
Q8VHH8	Il25	Q9JIP3	Il17rb	REC/CYT
Q8VHH8	Il25	Q60943	Il17ra	REC/CYT
Q8BVZ5	Il33	P14719	Il1rl1	REC/CYT
P07750	Il4	P16382	Il4ra	REC/CYT
P07750	Il4	O09030	Il13ra1	REC/CYT
P08505	Il6	P22272	Il6ra	REC/CYT
P08505	Il6	Q00560	Il6st	REC/CYT
P10168	Il7	P16872	Il7r	REC/CYT
P11672	Lcn2	Q9D9E0	Slc22a17	REC/CYT
P16045	Lgals1	Q07797	Lgals3bp	PPI
P16045	Lgals1	Q9DBX3	Susd2	REC/CYT
P16110	Lgals3	P16110	Ptprc	REC/CYT
P16110	Lgals3	Q62351	Tfrc	REC/CYT
P21956	Mfge8	P43406	Itgav	REC/CYT
P21956	Mfge8	O54890	Itgb3	REC/CYT
P21956	Mfge8	O70309	Itgb5	REC/CYT
Q64299	Ccn3	Q01705	Notch1	REC/CYT
Q64299	Ccn3	P11688	Itga5	REC/CYT
Q64299	Ccn3	Q61739	Itga6	REC/CYT
Q64299	Ccn3	P43406	Itgav	REC/CYT
Q64299	Ccn3	P09055	Itgb1	REC/CYT
Q64299	Ccn3	O70309	Itgb5	REC/CYT
Q64299	Ccn3	O54890	Itgb3	REC/CYT
P20033	Pdgfa	P26618	Pdgfra	REC/CYT
P49764	Pgf	P35969	Flt1	REC/CYT
Q62009	Postn	P43406	Itgav	REC/CYT
Q62009	Postn	O54890	Itgb3	REC/CYT
Q62009	Postn	O70309	Itgb5	REC/CYT
Q62009	Postn	P98063	Bmp1	PPI
P06879	Prl	Q08501	Prlr	REC/CYT
P48759	Ptx3	O08859	Tnfaip6	PPI
P48759	Ptx3	P98086	C1qa	PPI
P48759	Ptx3	P14106	C1qb	PPI
P48759	Ptx3	Q02105	C1qc	PPI
Q9DD06	Rarres2	P97468	Cmklr1	REC/CYT
Q9DD06	Rarres2	O35457	Ccrl2	REC/CYT
Q9DD06	Rarres2	Q8K087	Gpr1	REC/CYT

P10923	Spp1	P43406	Itgav	REC/CYT
P10923	Spp1	O54890	Itgb3	REC/CYT
P04202	Tgfb1	Q64729	Tgfbr1	REC/CYT
P04202	Tgfb1	Q62312	Tgfbr2	REC/CYT
P06804	Tnf	P25118	Tnfrsf1a	REC/CYT
P06804	Tnf	P25119	Tnfrsf1b	REC/CYT
O35235	Tnfsf11	O35305	Tnfrsf11a	REC/CYT
O35235	Tnfsf11	O08712	Tnfrsf11b	REC/CYT
Q7TS55	Tnfsf18	O35714	Tnfrsf18	REC/CYT
Q9JIE6	Tslp	P16872	Il7r	REC/CYT
Q9JIE6	Tslp	Q8CII9	Crlf2	REC/CYT
Q00731	Vegfa	P35969	Flt1	REC/CYT
Q00731	Vegfa	P35918	Kdr	REC/CYT
